# Supplementary material for: Chromatin‐bound cGAS is an inhibitor of DNA repair and hence accelerates genome destabilization and cell death
Source: EMBO J. 2019 Sep 23;38(21):e102718. doi: 10.15252/embj.2019102718 (PMC6826206; doi:10.15252/embj.2019102718)
Supplement: Supplementary file 1 — Appendix [file EMBJ-38-e102718-s001.pdf]

Jiang *et al.,* Chromatin-bound cGAS is an inhibitor of DNA repair and accelerates genome destabilization and cell death.

Appendix

Table of Contents **Page**

Table of Contents	Page
Appendix Figure S1	1
Appendix Figure S2	2
Appendix Figure S3	3
Appendix Table S1	4

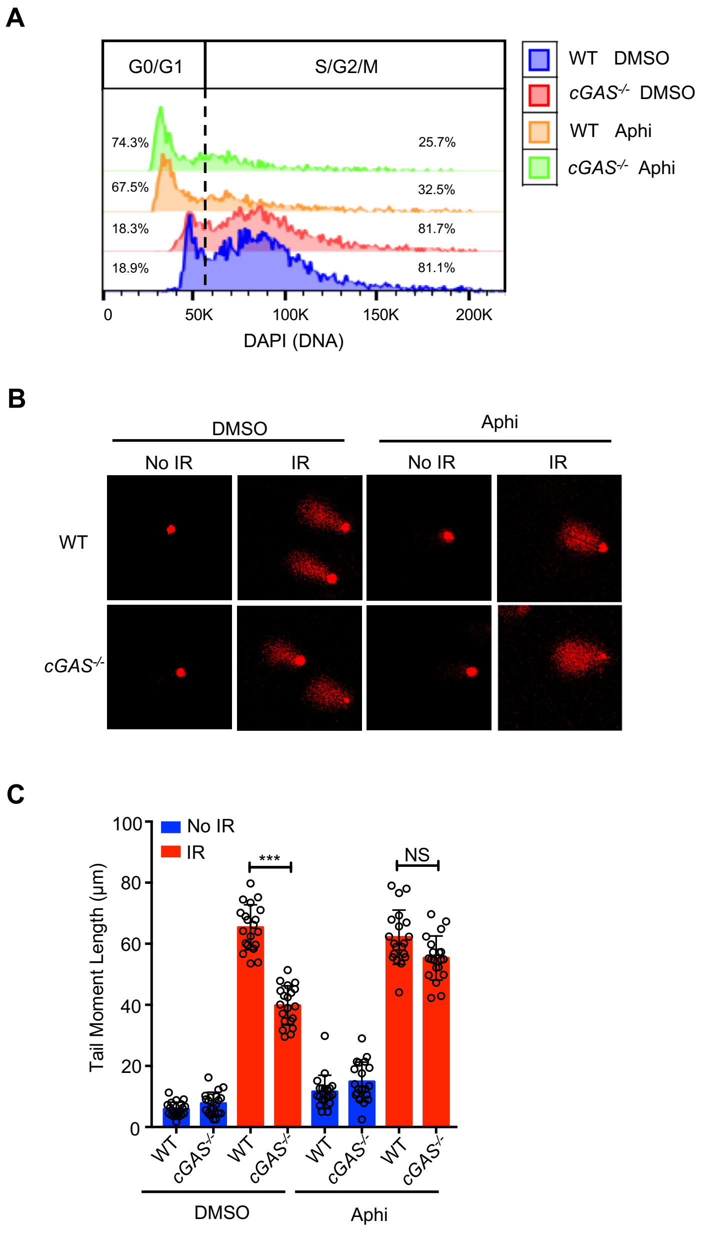

Appenix Figure S1.
cGAS suppress DNA repair active during S/G2 phase.

A. Flow cytometric cell cycle analysis of indicate cells treated with Aphidicolin(Aphi) or DMSO control.

B,C. cGAS does not suppress DNA repair in cells arrested at G1/ S boundary. Representative images (B) and corresponding quantification (C) of comet tails in WT and cGAS-/- BMDMos that were either treated with either DMSO (control), or Aphidicolin (+Aphi) then γ-irradiated (IR: 10 Gy) and analyzed 15 min later. Each data set in the graphs (C) represent mean score from 20 different fields with n > 200 comets.

Data information: Statistical significance was using one-way ANOVA followed by Sidak's post-test.. NS: P >0.05, ***P ≤ 0.001. experiments were repeated at least three times in triplicates. Data are represented as Mean ± SEM.

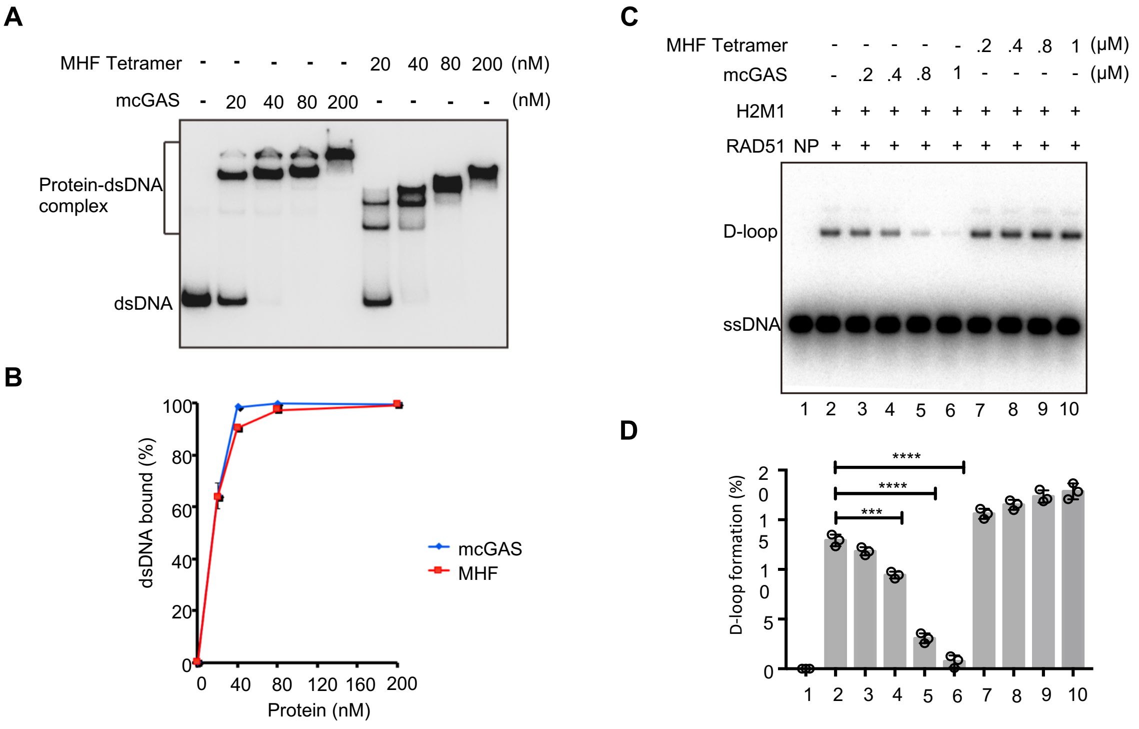

Appendix Figure S2.
Inhibition of D-loop formation is not a universal feature of all DNA binding proteins.

A,B. cGAS and MHF binds to dsDNA with similar affinity. 20-200 nM of cGAS or MHF were incubated with dsDNA at 37ºC and protein-dsDNA complexes analyzed by gel separation (A) densitometric quantification were graphed as the average of triplicates ± SD (B).

C. Pre-incubation of template dsDNA with cGAS blocks subsequent D-loop by RAD51 while pre-incubation with the DNA binding protein MHF does not.

D. Percentage of D-loop formed in each reaction (C) graphed as the average of triplicates ± SD.

Data information: Statistical significance was using one-way ANOVA followed by Sidak's post-test. ***P ≤ 0.001,****P ≤ 0.0001.

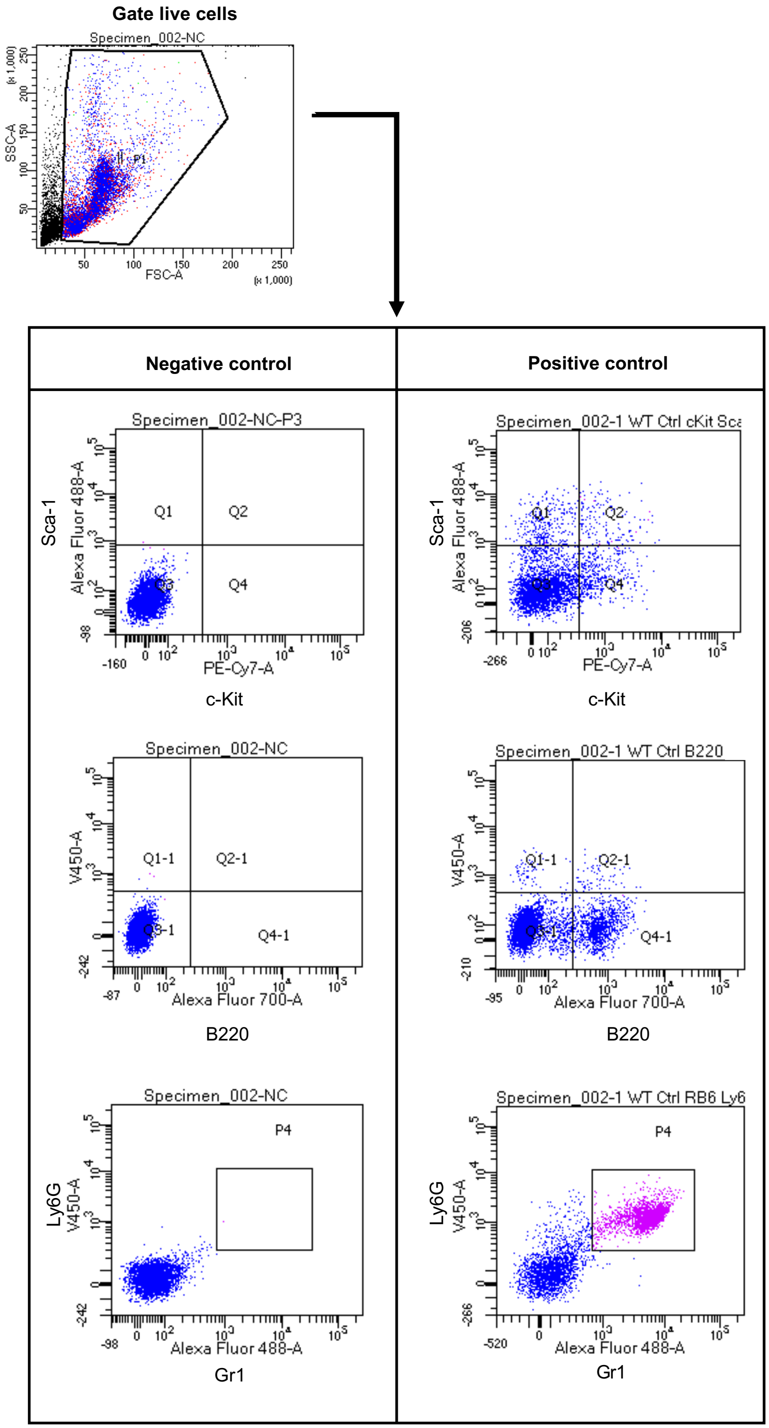

Appendix Figure S3.
Gating strategies for *in vivo* Bone marrow depletion.

Appendix Table S1. Oligonucleotides used in this study.

Name	Sequence	Assay
IFNB1	5’-CAGCAATTTTCAGTGTCAGAAGCT-3’<br>5’-TCATCCTGTCCTTGAGGCAGTA-3’	RT-PCR
18srRNA	5’-GATATGCTCATGTGGTGTTG-3’<br>5’- AATCTTCTTCAGTCGCTCCA-3’	RT-PCR
P1	TCTTATTTATGTCTCTTTTATTTCATTTCCTATATTTATTCCTATTATGT<br>TTTATTCATTTACTTATTCTTTATGTTCATTTTTTATATCCTTTACTTTA<br>TTTTCTCTGTTTATTCATTTACTTATTTTGTATTATCCTTATCTTATTTA	DNA strand exchange
P2	AGAATAAGTAAATGAATAAAACATAATAGGAATAAATA<br>TAGGAAATGAAATAAAAGAGAC	DNA strand exchange
P3	GTCTCTTTTATTTCATTTCCTATATTTATTCCTATTATG<br>TTTTATTCATTTACTTATTCT	DNA strand exchange
