# Supplementary material for: Chromatin‐bound cGAS is an inhibitor of DNA repair and hence accelerates genome destabilization and cell death
Source: EMBO J. 2019 Sep 23;38(21):e102718. doi: 10.15252/embj.2019102718 (PMC6826206; doi:10.15252/embj.2019102718)
Supplement: Supplementary file 2 — Expanded View Figures PDF [file EMBJ-38-e102718-s002.pdf]

Expanded View Figures

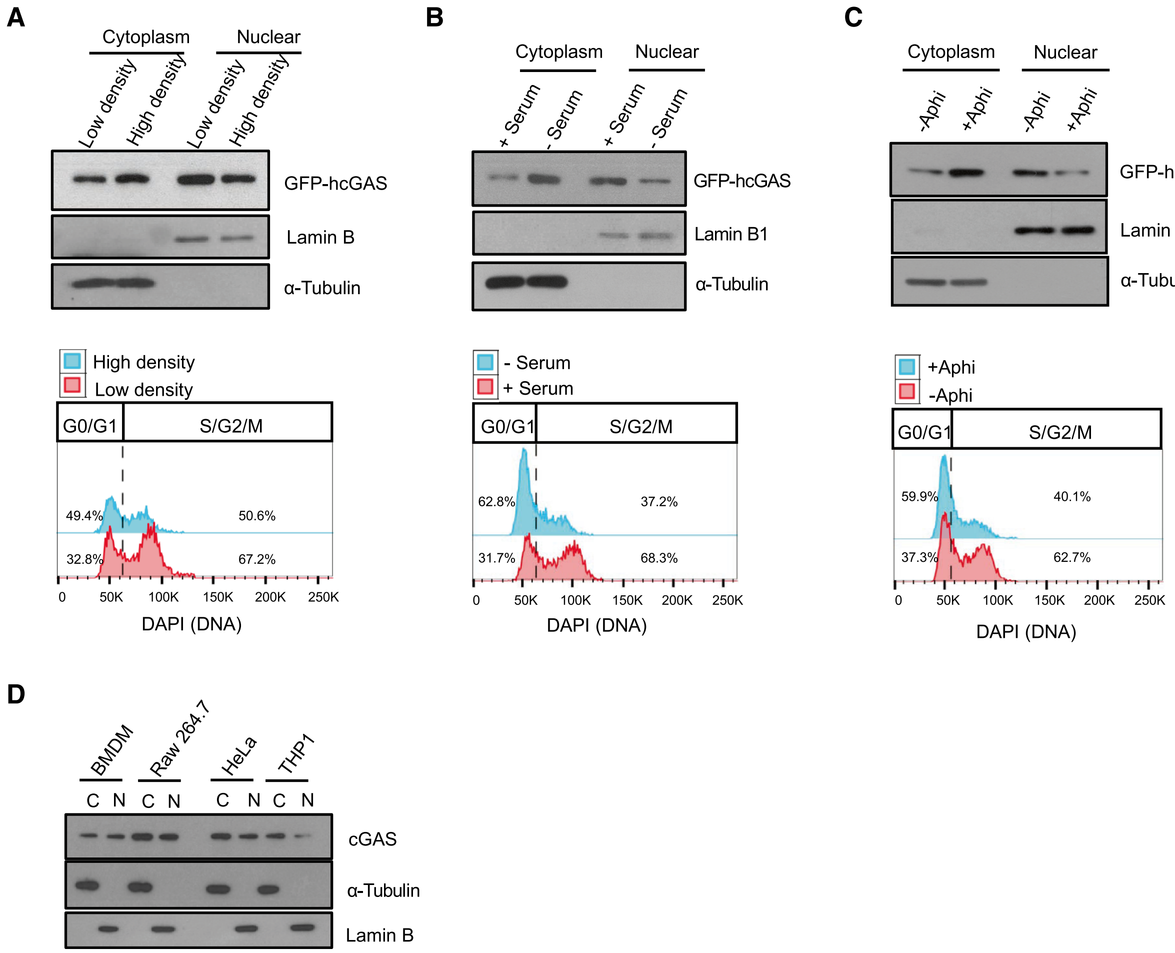

Figure EV1. cGAS is constantly present in the cytosol and nucleus.

A Immunoblot estimation of GFP-hcGAS in nuclear/cytosolic fractions and corresponding flow cytometric analysis of cell cycle of HEK293 cells cultured in low or high density. Lamin B and α-tubulin are nuclear and cytosolic markers, respectively.

B Immunoblot estimation of GFP-hcGAS in nuclear/cytosolic fractions and corresponding flow cytometric analysis of cell cycle of HEK293 cells cultured with or without serum. Lamin B and α-tubulin are nuclear and cytosolic markers, respectively.

C Immunoblot estimation of GFP-hcGAS in nuclear/cytosolic fractions and corresponding flow cytometric analysis of cell cycle of HEK293 cells cultured with or without aphidicolin. Lamin B and α-tubulin are nuclear and cytosolic markers, respectively.

D cGAS in nuclear/cytosolic fractions of indicated cell types.

Source data are available online for this figure.

 © 2019 The Authors

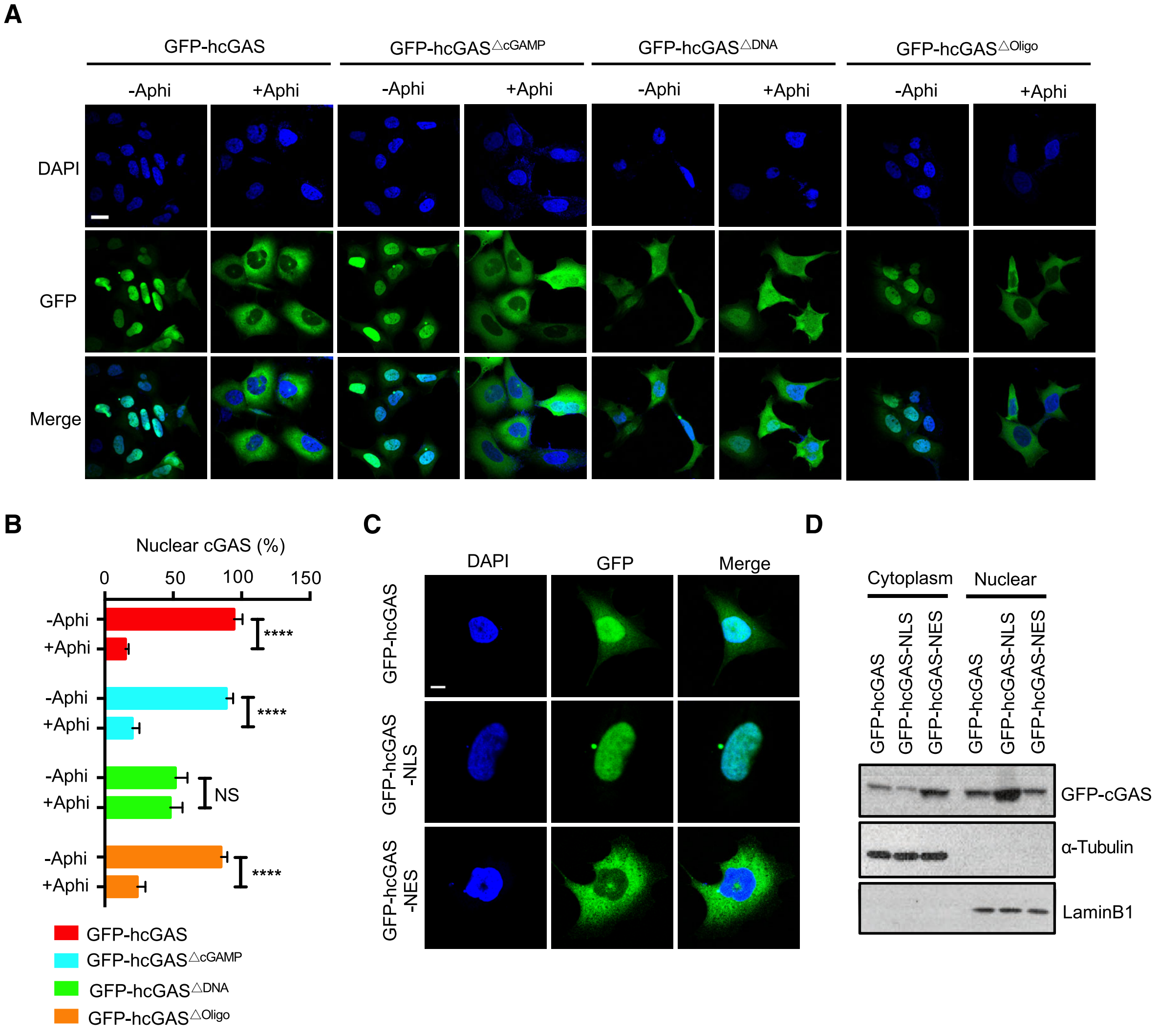

Figure EV2. Localization and retention of cGAS in the nucleus is due to is avid binding to DNA.

A Fluorescence images of GFP-hcGAS, GFP-hcGASΔcGAMP, GFP-hcGASΔDNA, and GFP-hcGASΔOligo in HEK293 cells cultured with or without aphidicolin. Scale bar: 20 μm.

B Corresponding quantification of (A). The nuclear cGAS/total cGAS was calculated from 6 different fields with $n > 50$ cells.

C, D A nuclear export signaling (NES) is not sufficient to dislodge chromatin-bound cGAS from the nucleus. (C) Fluorescence images of GFP-hcGAS, GFP-hcGAS-NLS, and GFP-hcGAS-NES in HEK293 cells. Scale bar: 10 μm. (D) Immunoblots of subcellular fractions of GFP-hCGAS-, GFP-hcGAS-NLS-, and GFP-hCGAS-NES-expressing HEK293 cells.

Data information: Data are presented as means ± SEM. Statistical significance was assessed using one-way ANOVA followed by Sidak's post-test. NS: $P > 0.05$ and ****$P \leq 0.0001$.
Source data are available online for this figure.

© 2019 The Authors

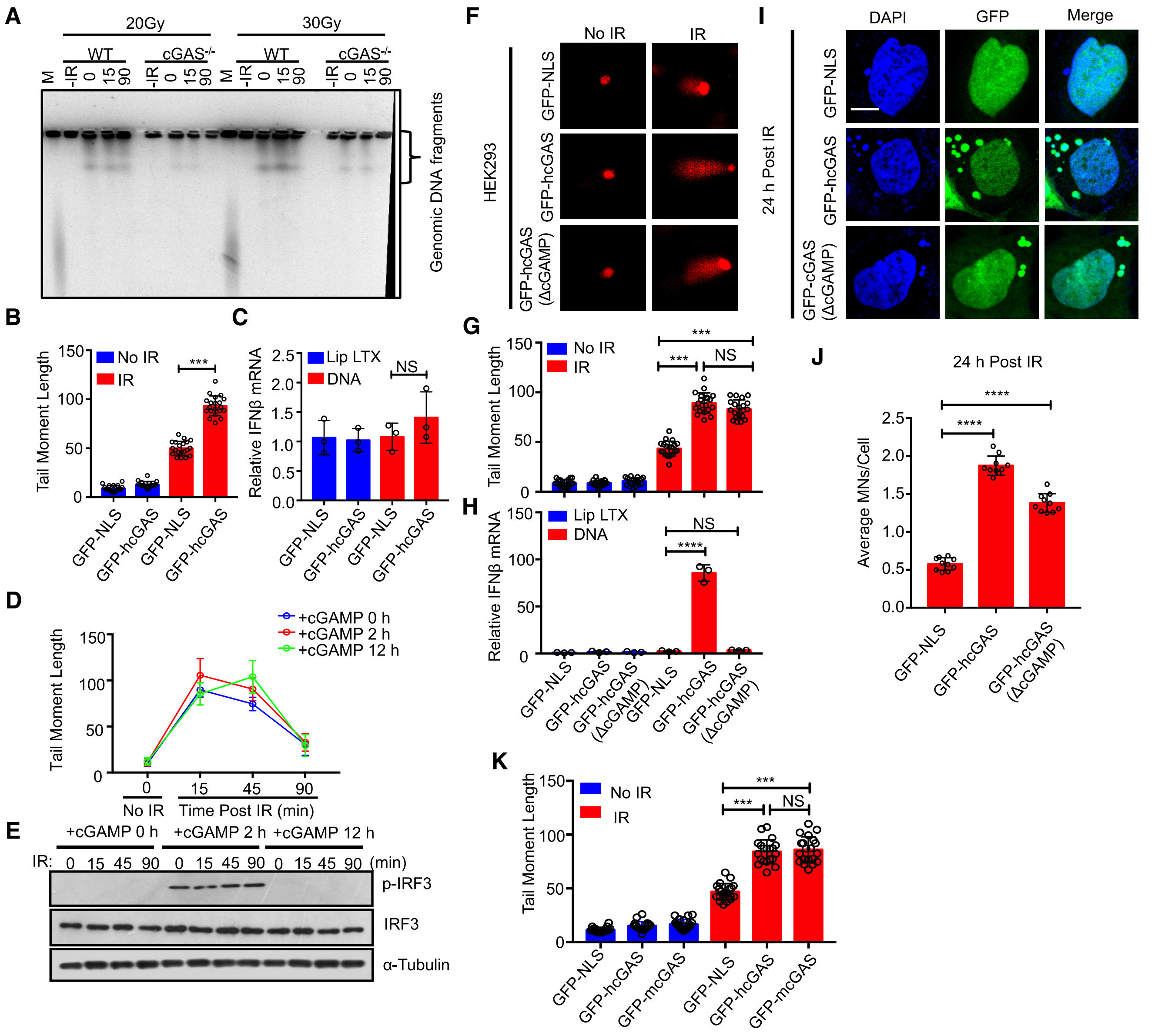

Figure EV3. STING signaling is dispensable for inhibition of DNA repair by cGAS.

A Pulsed-field gel electrophoresis analysis of γ-irradiated (10 Gy) WT and cGAS$^{-/-}$ BMDMos.

B, C Comet assay in GFP-NLS- and GFP-hcGAS-expressing HEK293T cells γ-irradiated (IR: 10 Gy) for 15 min (B). RT–PCR analysis of IFNB1 response in GFP-NLS- or GFP-hcGAS-expressing HEK293T cells stimulated with transfected DNA for 6 h (C).

D, E Comet assay of HEK293 cells stimulated with 10 μg/ml cGAMP for indicate periods, then γ-irradiated and incubated at 37°C for indicated duration (D). (E) Immunoblots of IRF3 phosphorylation in HEK293 cells treated as in (D).

F–H Images (F) and quantifications (G) of comet tails 15 min after irradiation of GFP-NLS-, GFP-hcGAS-, and GFP-hcGASΔcGAMP-expressing HEK293 cells. RT–PCR analysis of IFNB1 response in GFP-NLS- or GFP-hcGAS-expressing HEK293 cells stimulated with transfected 23 DNA for 6 h (H).

I, J Images (I) and quantifications (J) of micronuclei in GFP-NLS- and GFP-hcGASΔcGAMP-expressing HEK293 cells 24 h after γ-irradiation (IR; 10 Gy). DAPI (DNA). Scale bar: 10 μm. Each data set bar comet graph was calculated from six different microscopic fields with over 200 cells.

K Quantifications of comet tails 15 min after irradiation (10 Gy) of GFP-NLS-, GFP-hcGAS-, or GFP-mcGAS-expressing HEK293 cells. Each data set bar comet graph was calculated from six different microscopic fields with over 200 cells.

Data information: Statistical significance was assessed using one-way ANOVA followed by Sidak's post-test. NS $P > 0.05$, ***$P \leq 0.001$, and ****$P \leq 0.0001$. Mean ± SEM of $n = 3$ independent experiments.

Source data are available online for this figure.

 © 2019 The Authors

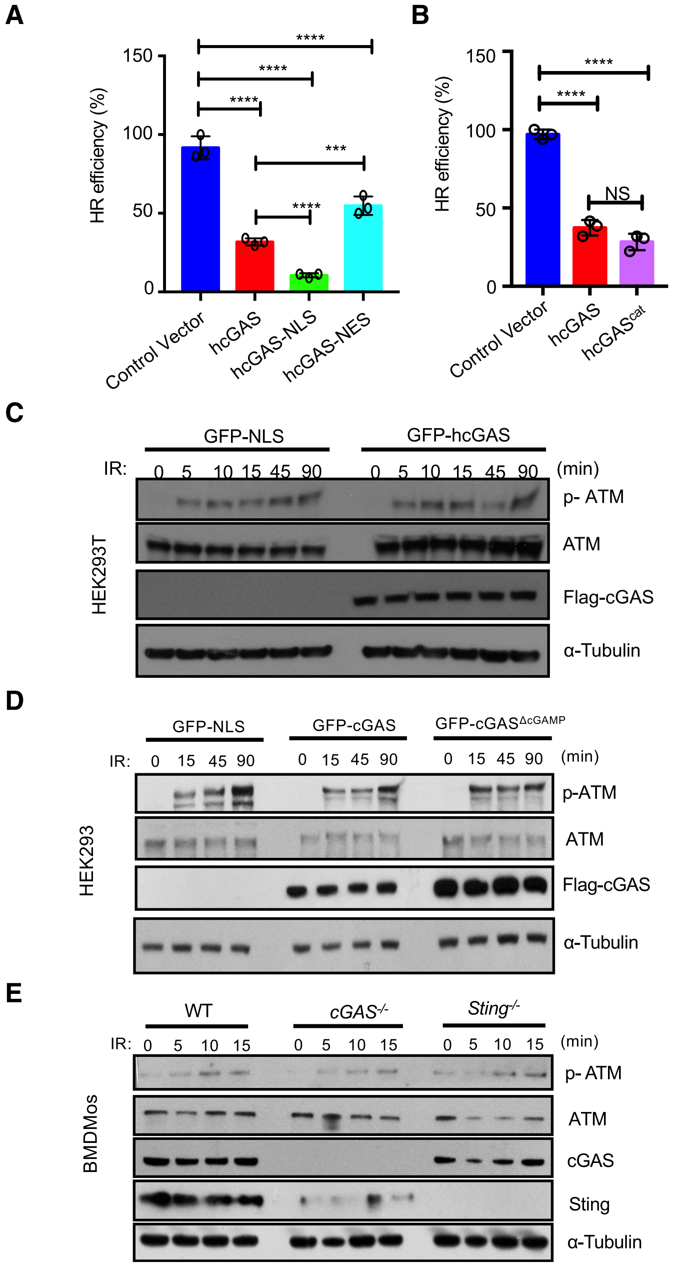

Figure EV4. cGAS suppresses DNA repair without inhibiting ATM activation.

A Reporter assays showing the effect of NLS and NES on cGAS-mediated inhibition of DNA repair.

B Both full-length hcGAS and hcGAS^{cat} (161–522aa) inhibit HR repair.

C–E cGAS does not impede ATM activation. ATM phosphorylation in γ-irradiated (10 Gy) GFP-NLS- and GFP-hcGAS-expressing HEK293T cells (C), GFP-NLS-, GFP-hcGAS-, and GFP-hcGASΔcGAMP-expressing HEK293 cells (D), or γ-irradiated (2.5 Gy) WT, cGAS$^{-/-}$, and Sting$^{-/-}$ BMDMos (E).

Data information: Data are means ± SD, n = 3. Statistical significance was assessed using one-way ANOVA followed by Sidak's post-test. ***P < 0.001 ****P < 0.0001, NS: P > 0.05.

Source data are available online for this figure.

© 2019 The Authors

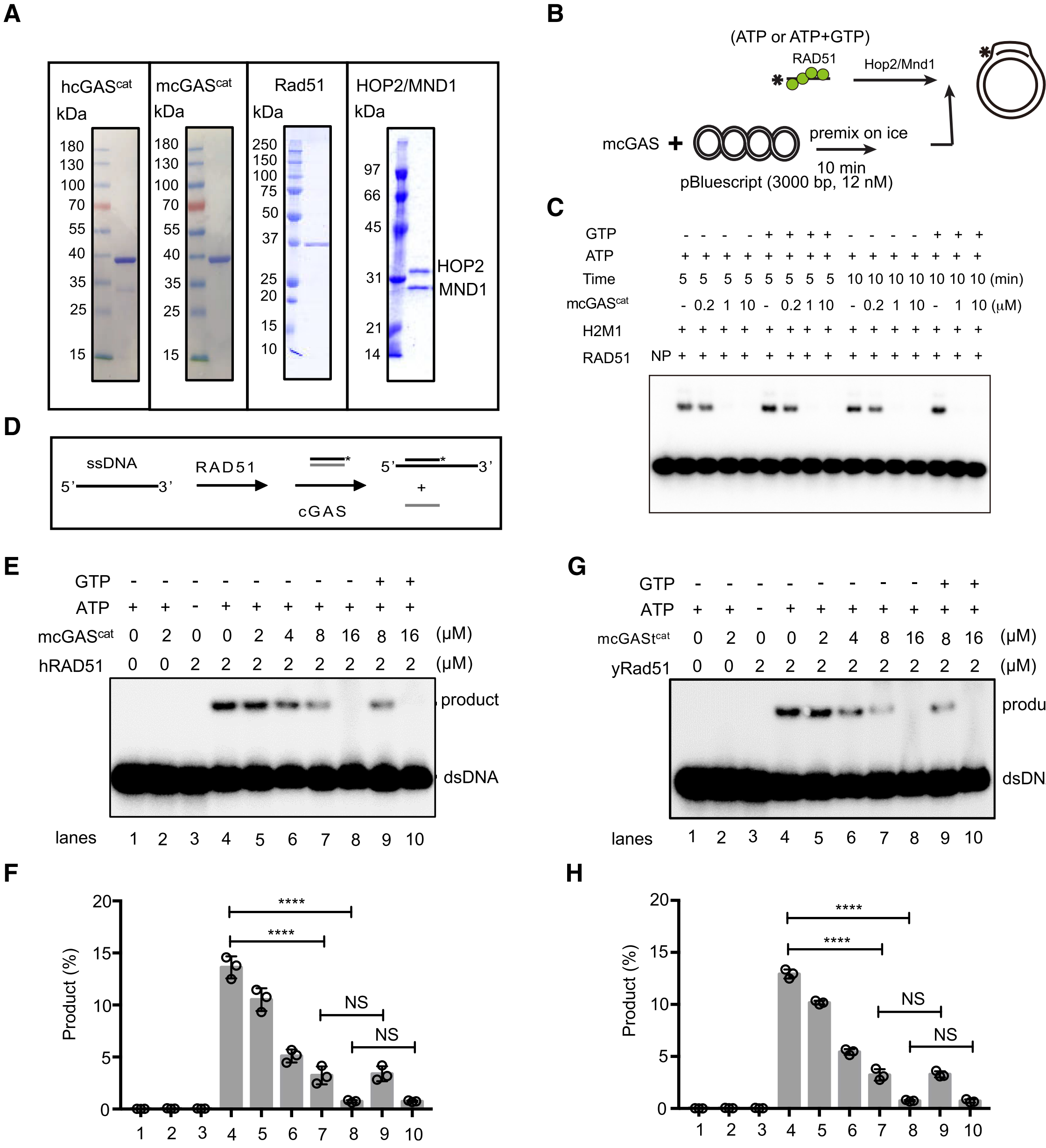

Figure EV5. cGAS inhibits RAD51-mediated DNA strand exchange and D-loop formation.

A Coomassie Blue staining of purified hcGAS^{cat}, mcGAS^{cat}, Rad51, HOP2, and MND1.

B Schematics of the D-loop assay.

C Pre-incubation of template dsDNA with cGAS blocks subsequent D-loop formation regardless of the presence of cGAMP precursors (ATP+GTP).

D Schematics of the strand exchange reaction.

E–H Pre-incubation of dsDNA with cGAS protein inhibited the DNA strand exchange activity of human RAD51 (E, F) and yeast Rad51 (G, H) regardless of the presence of precursors (ATP+GTP) of cGAMP. The percentage of DNA strand exchange in each reaction was graphed as the average of triplicates ± SD.

Data information: Statistical significance was assessed using one-way ANOVA followed by Sidak's post-test. NS: $P > 0.05$, ****$P \leq 0.0001$.
Source data are available online for this figure.

 © 2019 The Authors
