# Supplementary figures and images for: Chromatin‐bound cGAS is an inhibitor of DNA repair and hence accelerates genome destabilization and cell death
Source: EMBO J. 2019 Sep 23;38(21):e102718. doi: 10.15252/embj.2019102718 (PMC6826206; doi:10.15252/embj.2019102718)

Figure EV1A

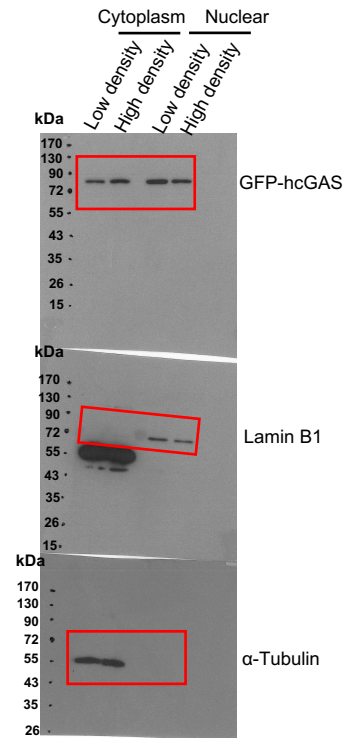

Figure EV1B

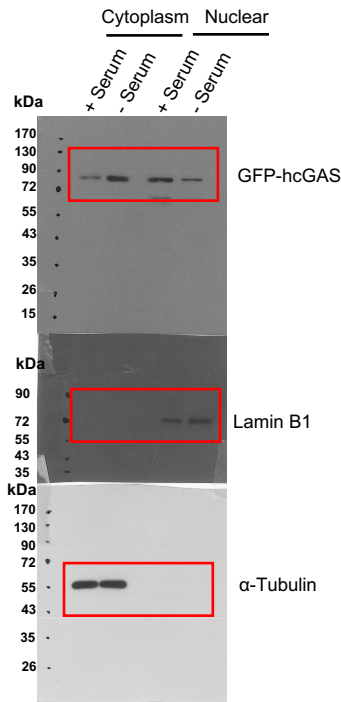

Figure EV1C

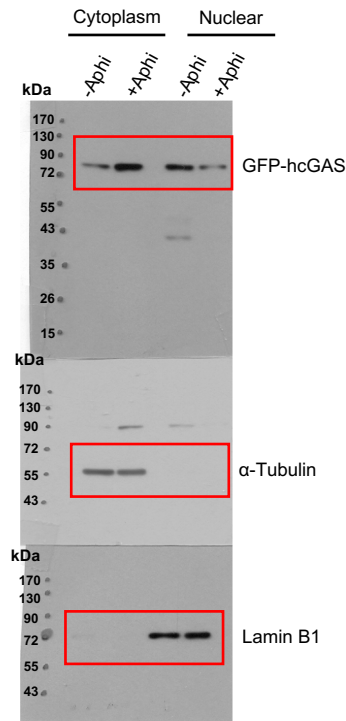

Figure EV1D

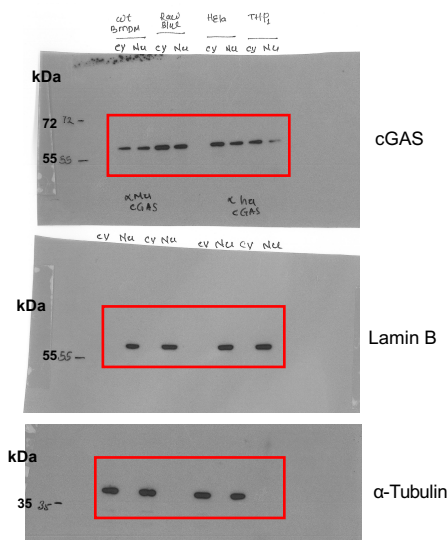

Supplement: Supplementary file 3 — Source Data for Expanded View [file EMBJ-38-e102718-s010.zip › Source_Data_for_Figure_EV1.pdf]

**Figure EV3F**

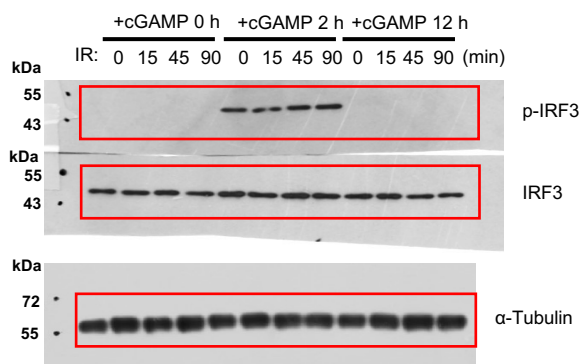

Figure EV3F

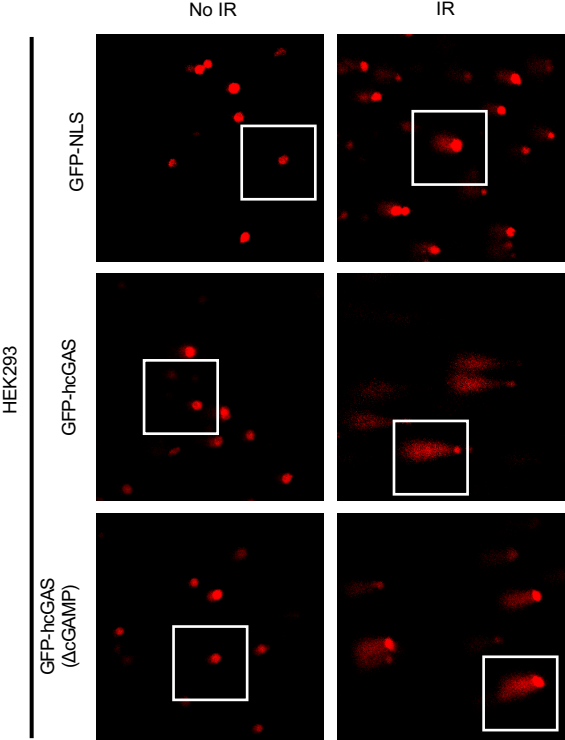

Figure EV3I

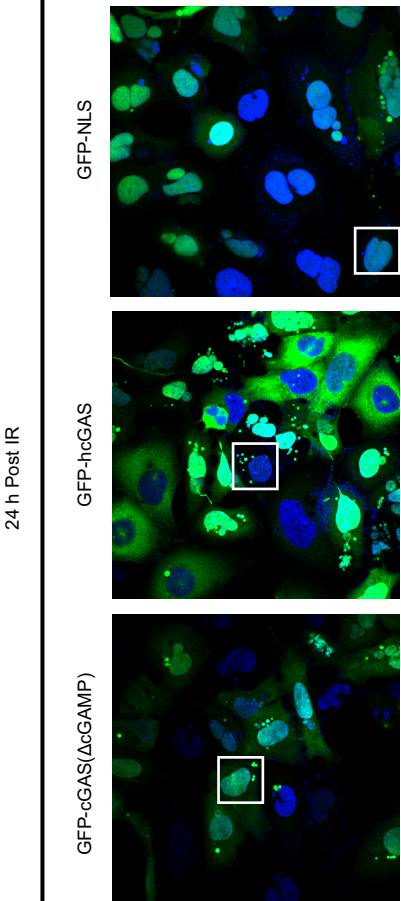

Supplement: Supplementary file 3 — Source Data for Expanded View [file EMBJ-38-e102718-s010.zip › Source_Data_for_Figure_EV3.pdf]

Figure EV2D

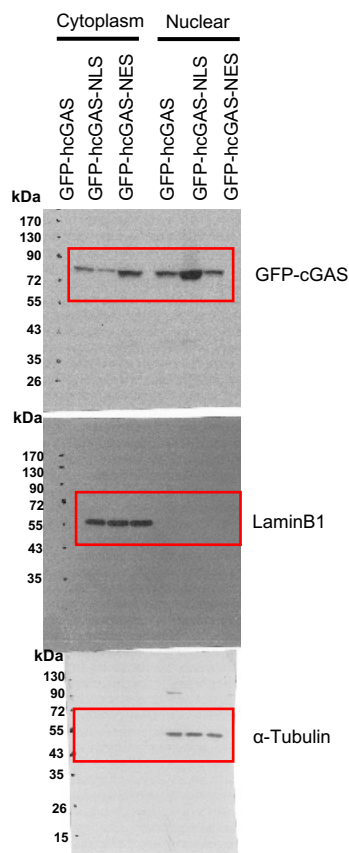

Figure EV2A is already the Raw data

Figure EV2C

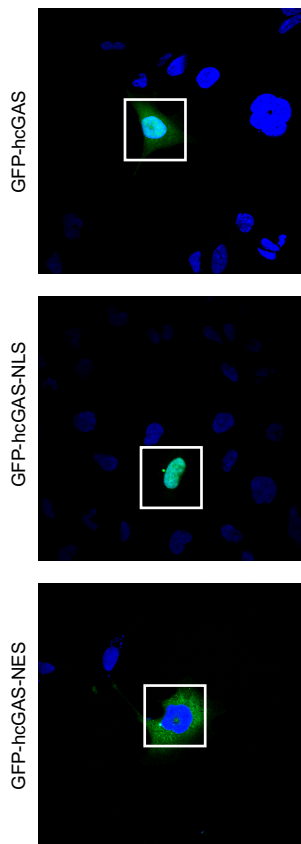

Supplement: Supplementary file 3 — Source Data for Expanded View [file EMBJ-38-e102718-s010.zip › Source_Data_for_Figure_EV2.pdf]

Figure EV5C

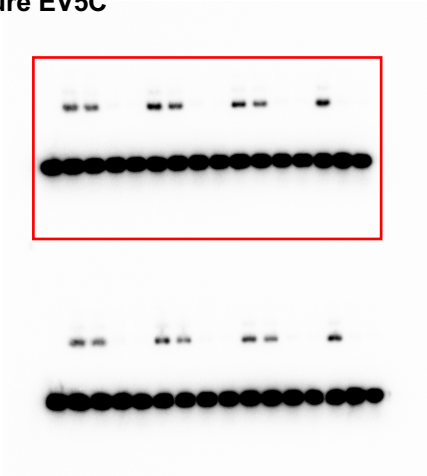

Figure EV5E

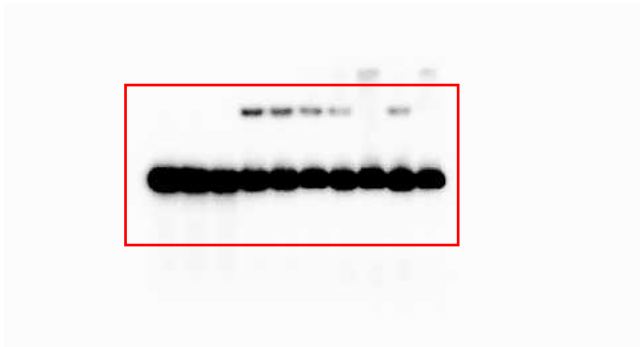

Figure EV5G

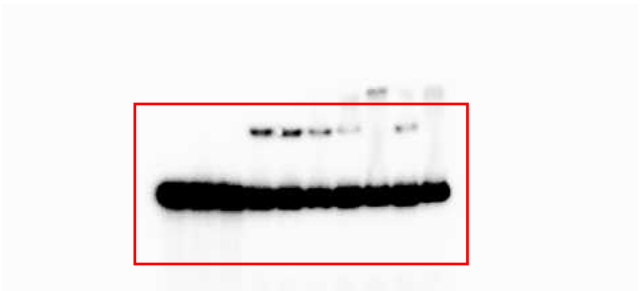

Supplement: Supplementary file 3 — Source Data for Expanded View [file EMBJ-38-e102718-s010.zip › Source_Data_for_Figure_EV5.pdf]

Figure EV4C

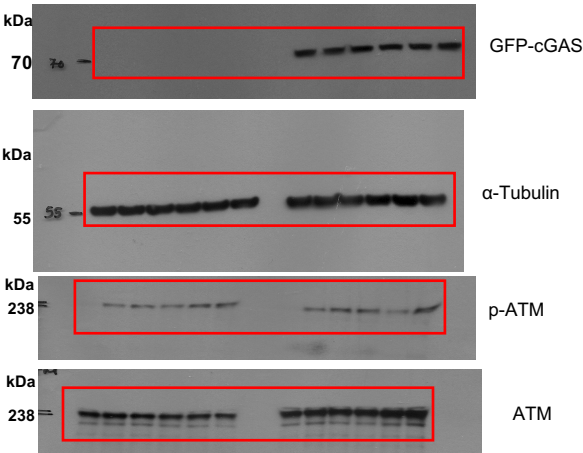

Figure EV4D

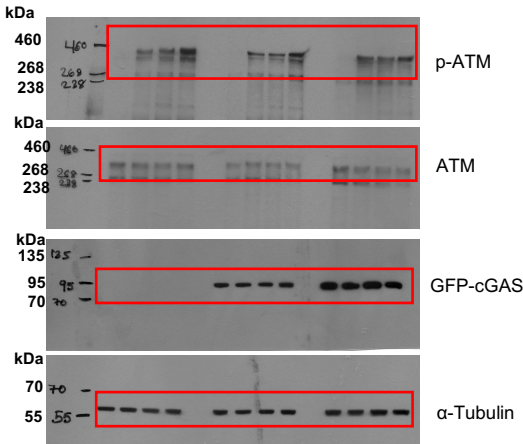

Figure EV4E

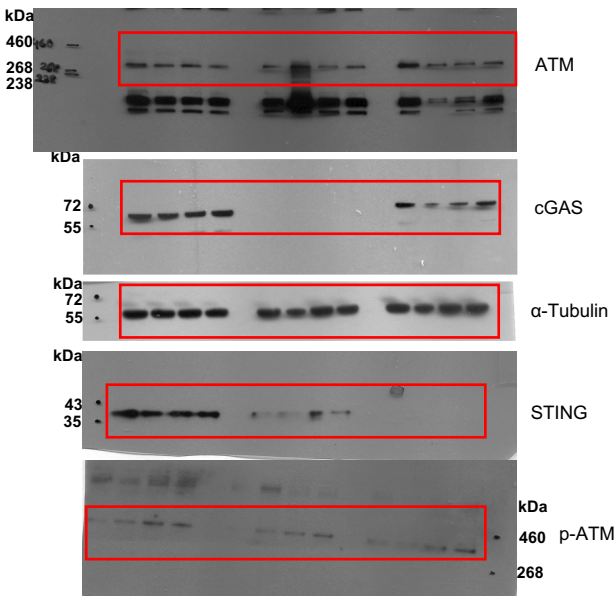

Supplement: Supplementary file 3 — Source Data for Expanded View [file EMBJ-38-e102718-s010.zip › Source_Data_for_Figure_EV4.pdf]

Figure 1D

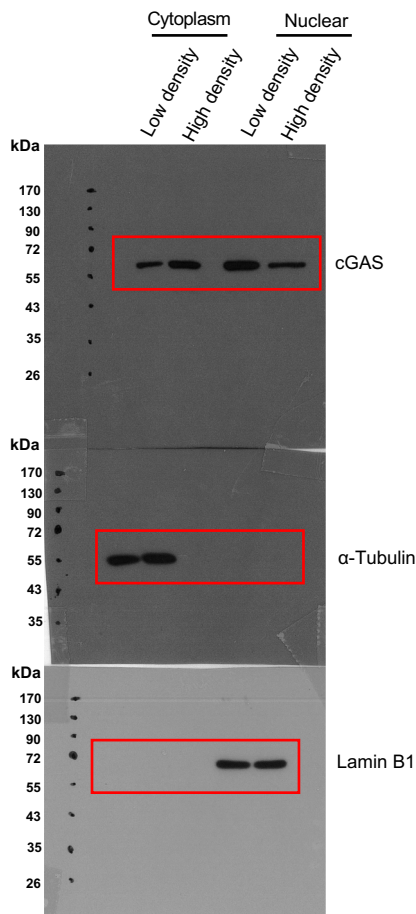

Figure 1E

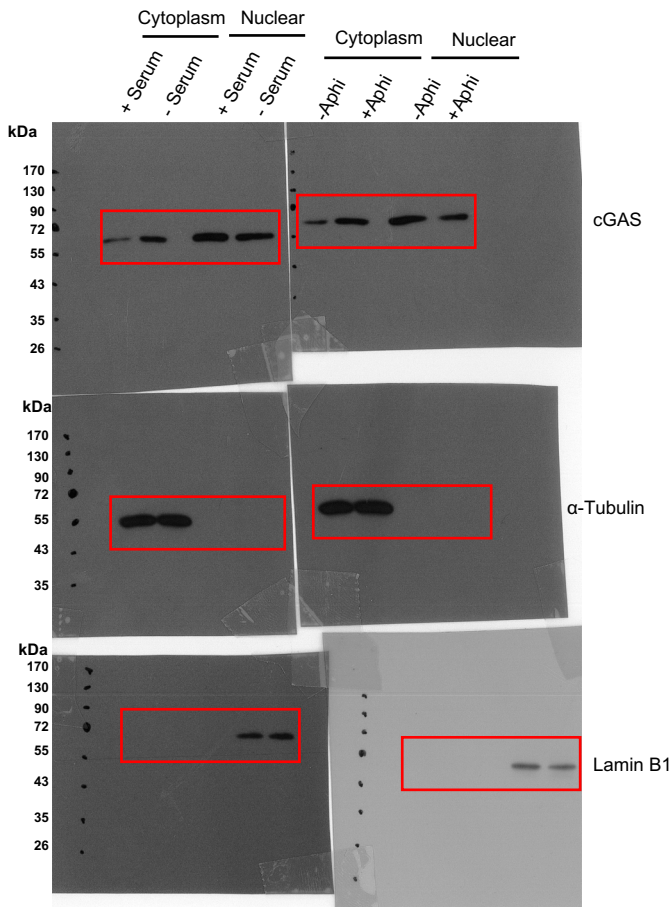

Figure 1F

Figure 1A

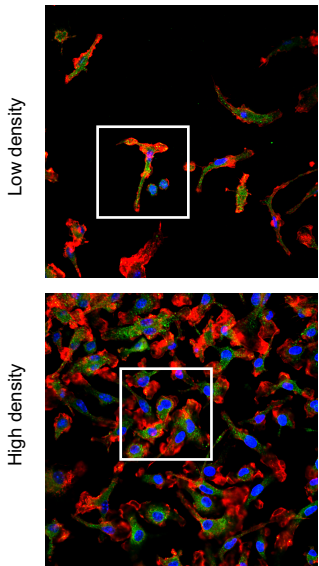

Figure 1B

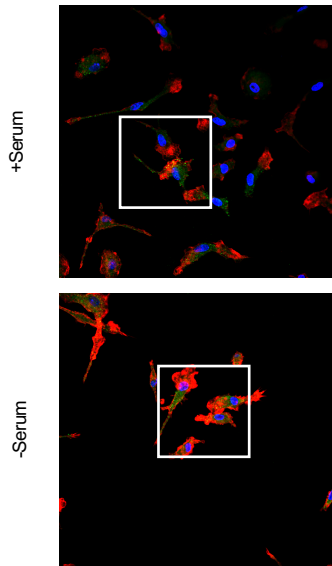

Figure 1C

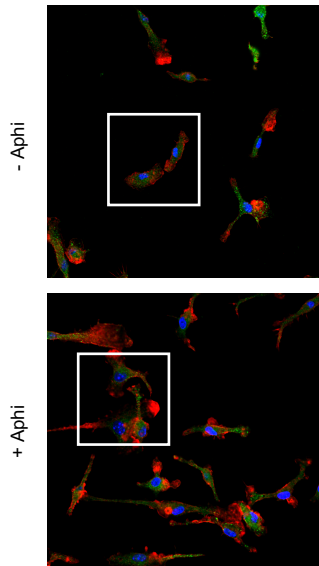

Supplement: Supplementary file 5 — Source Data for Figure 1 [file EMBJ-38-e102718-s003.pdf]

Figure 2A

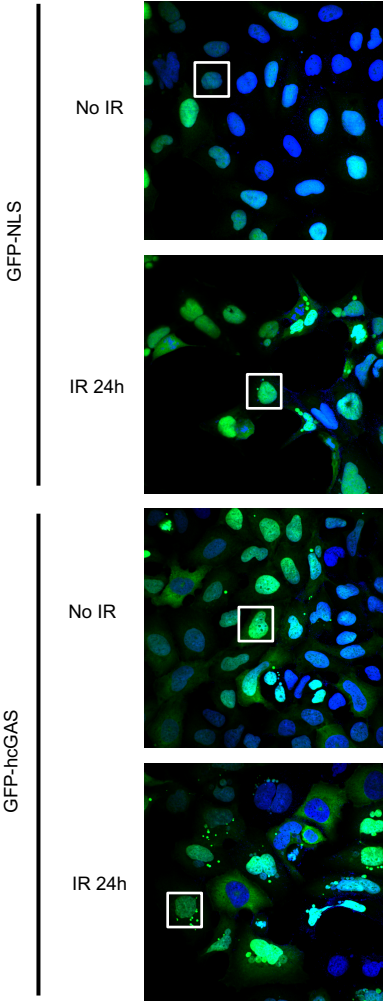

Figure 2E

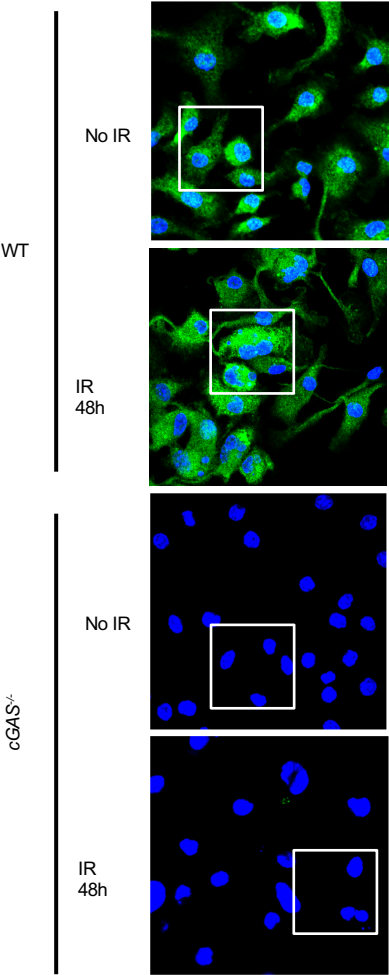

Supplement: Supplementary file 6 — Source Data for Figure 2 [file EMBJ-38-e102718-s004.pdf]

Figure 3A

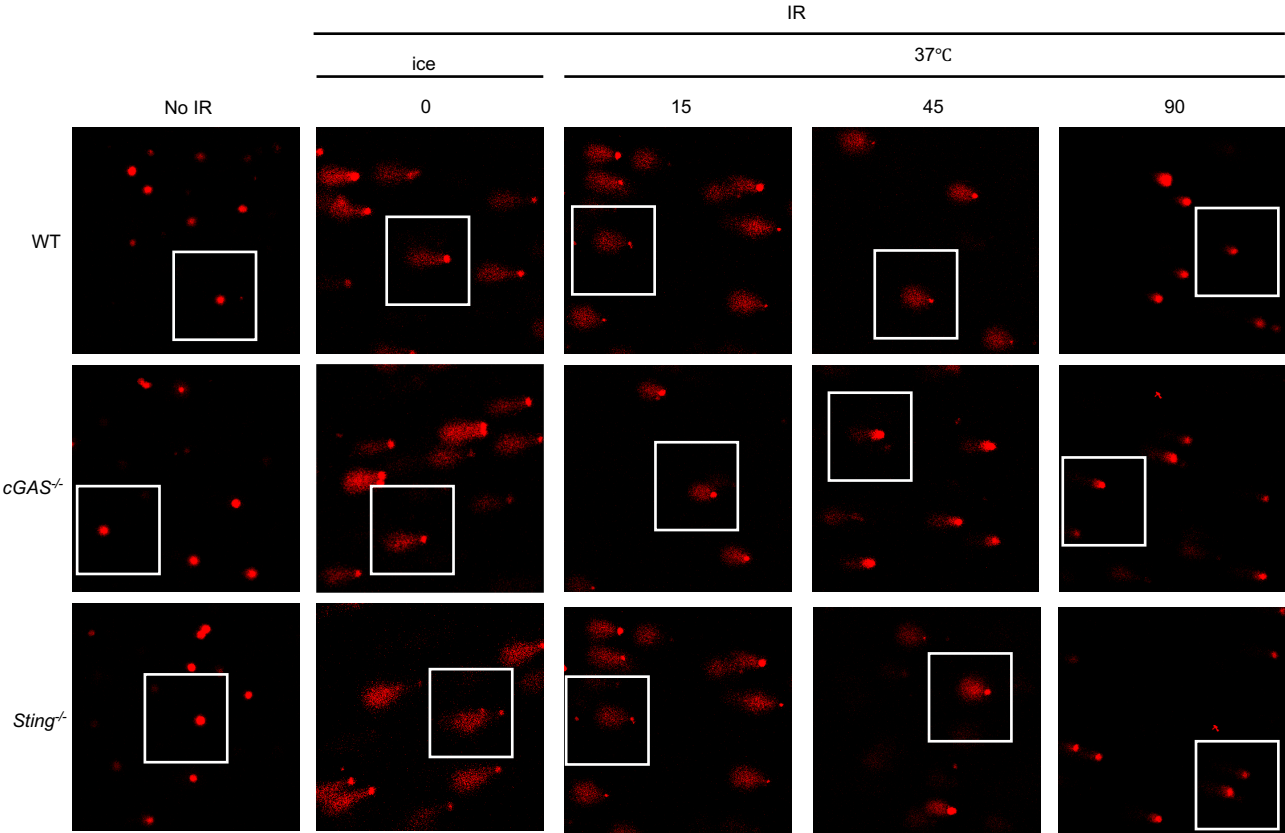

Figure 3C

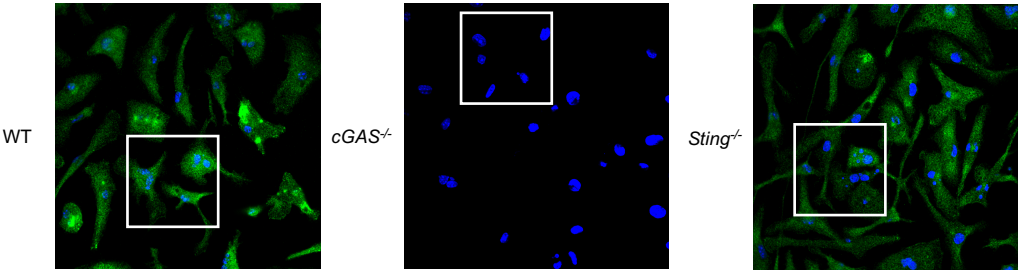

Supplement: Supplementary file 7 — Source Data for Figure 3 [file EMBJ-38-e102718-s005.pdf]

Figure 5C

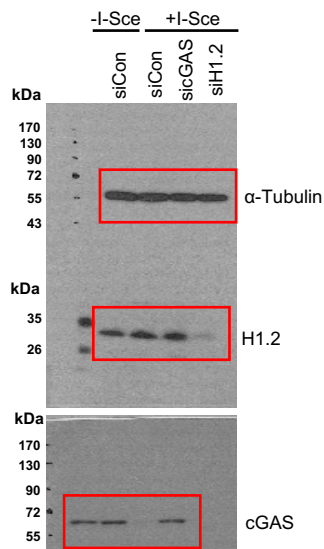

Figure 5D

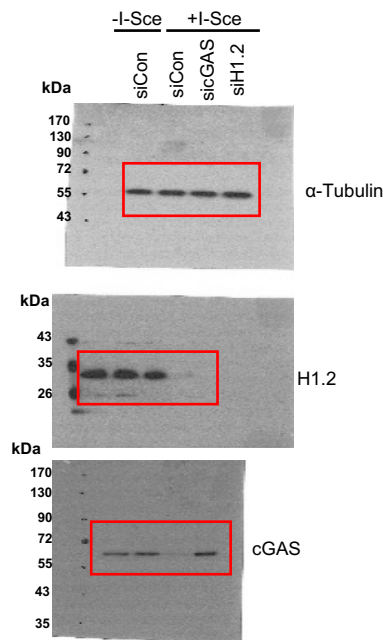

Figure 5E,F

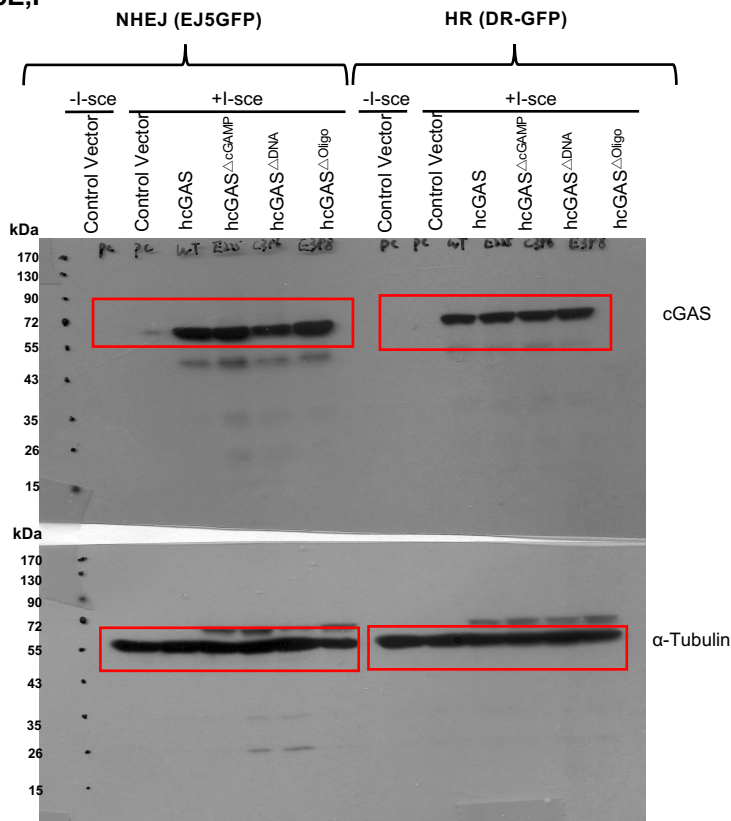

Supplement: Supplementary file 8 — Source Data for Figure 5 [file EMBJ-38-e102718-s006.pdf]

Figure 6C

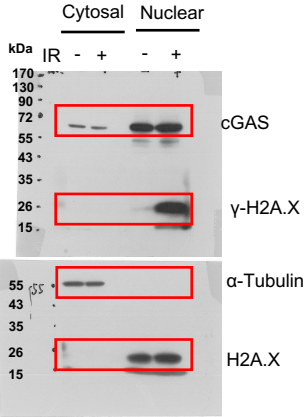

Figure 6D

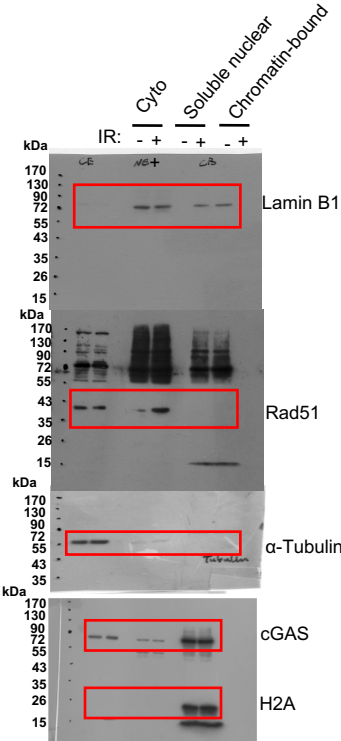

Figure 6E

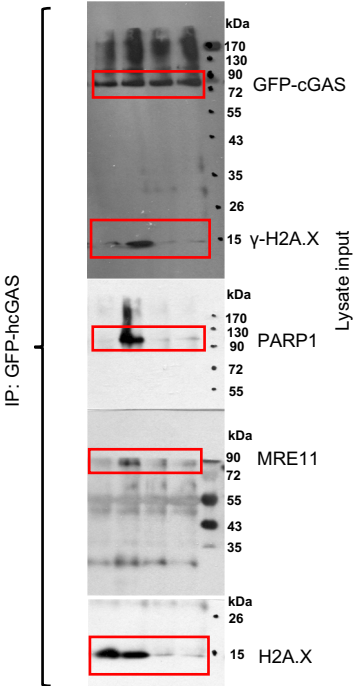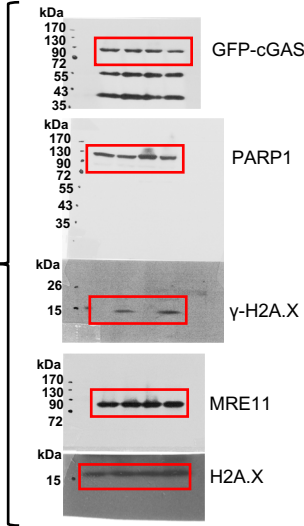

Figure 6G

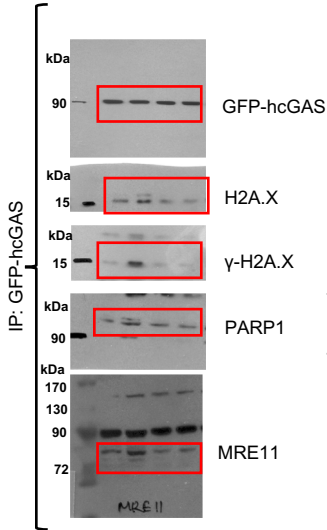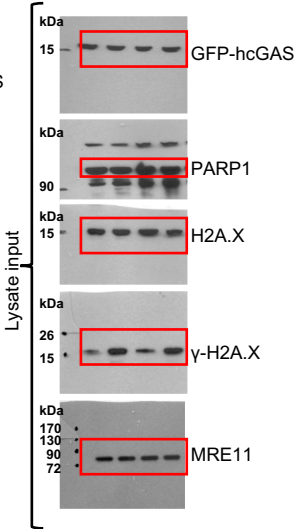

Figure 6A

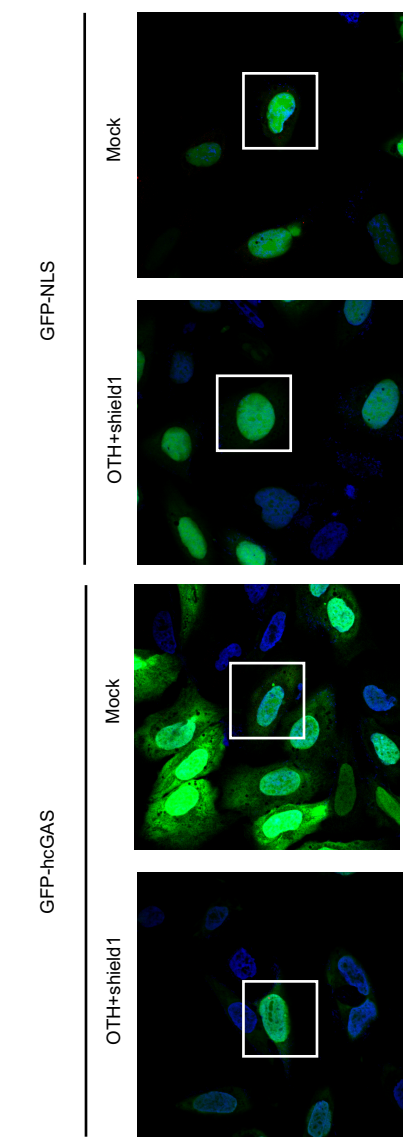

Figure 6B

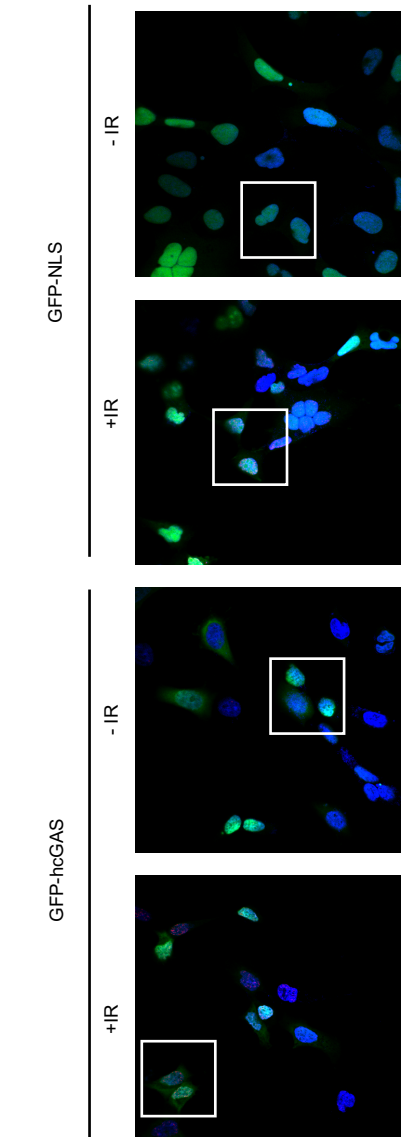

Supplement: Supplementary file 9 — Source Data for Figure 6 [file EMBJ-38-e102718-s007.pdf]

Figure 7C

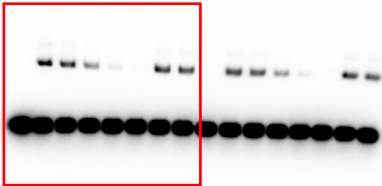

Figure 7E

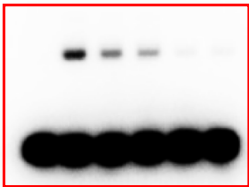

Figure 7A

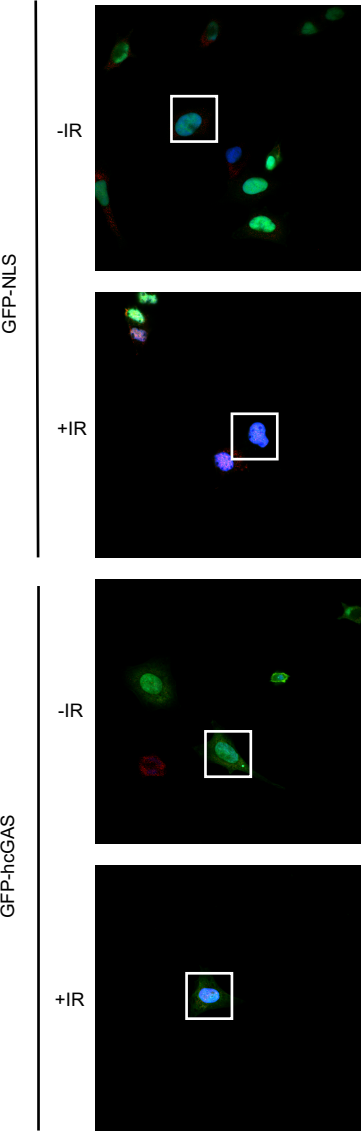

Supplement: Supplementary file 10 — Source Data for Figure 7 [file EMBJ-38-e102718-s008.pdf]

Figure 8A,H

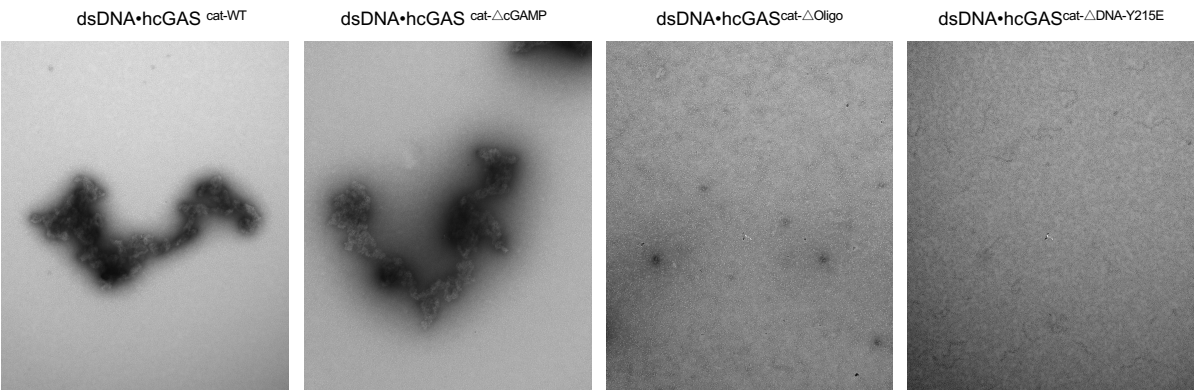

**Figure 8B**

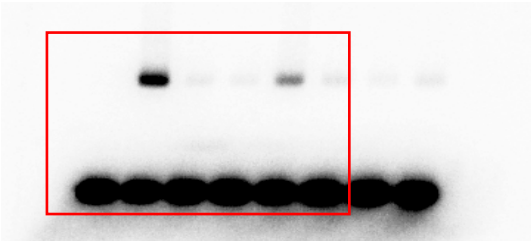

**Figure 8I**

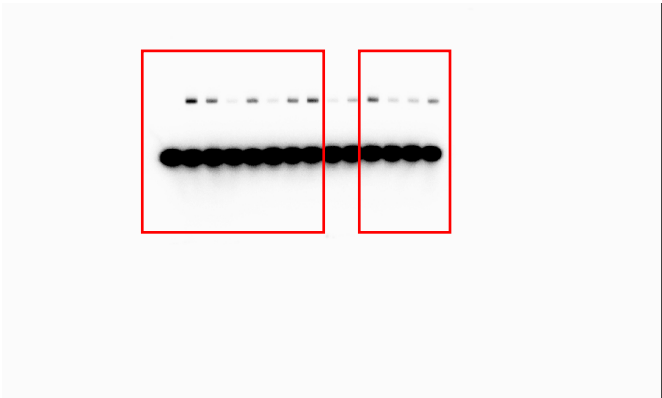

Supplement: Supplementary file 11 — Source Data for Figure 8 [file EMBJ-38-e102718-s009.pdf]
